# Student, Teacher, and School Counselor Perceptions of National School Uniforms in Malaysia

**DOI:** 10.3389/fpsyg.2020.01871

**Published:** 2020-07-28

**Authors:** Jhia Mae Woo, Cai Lian Tam, Gregory B. Bonn, Brendon Tagg

**Affiliations:** ^1^Department of Psychology, Monash University Malaysia, Subang Jaya, Malaysia; ^2^Department of Global and Social Studies, King Fahd University of Petroleum and Minerals, Dhahran, Saudi Arabia

**Keywords:** national school uniform, school uniform perception, student morale, national identity, school identity

## Abstract

The national school uniform remained a requirement for all primary and secondary school students in Malaysia since its first introduction by the British missionaries in the nineteenth century. Although it is commonly thought that wearing uniforms improve cohesion among students, little research has been done to investigate the perception of national school uniforms and perceived cohesion among Malaysian students. The aim of the current study is to explore the experiences of former students, teachers and school counselors in relation to Malaysia’s national school uniform policy, their influence on student life, and their relationship to school and national cohesion. Three studies were conducted. In Study 1, Malaysian secondary school graduates (*N* = 192) completed a self-report questionnaire with close and open-ended questions. In Study 2 and study 3, secondary school teachers (*N* = 10) and school counselors (*N* = 6) participated in semi-structured interviews, respectively. Although students felt the uniforms were unattractive, they had neutral to somewhat positive feelings about them overall. They did not report greater school cohesion or national identity related to uniforms but did report that uniforms lessened the importance of socioeconomic and other differences. Teachers and counselors reported similar views; they did not perceive much influence on national or school identity but did see advantages in terms of discipline and the lessening of student inequality. Generally, teachers and counselors were in favor of school uniforms but some suggested that uniform designs could be more culturally inclusive, particularly in regard to Muslim and non-Muslim students. Limitations of using non-representative sampling and future direction for Malaysian school uniforms were discussed. This study highlights the important characteristics that should be taken into consideration by educators and policymakers for future improvement of the national school uniform policy in Malaysia.

## Introduction

As part of a national school uniform policy, all primary and secondary school students in Malaysia are required to wear a standardized uniform when attending school. Although specific badges identify the school that each student attends, and there are different versions for males as well as Muslim and non-Muslim females, students all over the country are required to wear essentially the same outfits. This study explores this policy from the perspectives of students, teachers, and school counselors with experience in the Malaysian secondary school system.

### The Utility of School Uniforms

The national school uniform in Malaysia is considered, by government policy, a means of establishing national identity (The Star, 2010). Proponents of school uniforms outside Malaysia have argued that they promote feelings of belonging and self-esteem (Mahlangu, 2017), as well as limit some types of disciplinary and behavioral problems (Baumann and Hana, 2016). Generally, uniforms are thought of as a means of establishing conformity and institutional affiliation (Craik, 2003), with standardized apparel alleviating many appearance-related concerns, lessening self-consciousness and reducing some forms of competitiveness (Wade and Stafford, 2003). Perhaps relatedly, some findings have connected uniforms to improved academic performance and attendance (Gentile and Imberman, 2012; Baumann and Hana, 2016). Not surprisingly, school uniforms also have their opponents. While school uniforms obviously standardize appearance, studies in the United States and Korea have suggested that this does not necessarily relate to improved belonging (Brunsma, 2006; Park, 2013). Generally, students are observed to individuate themselves and compete even within the limited structures of uniform codes.

Although Malaysia has a national school uniform policy, a relatively uncommon phenomenon throughout the world, to date, little research has looked at its pros and cons. Although there are minor differences between schools, essentially, all students throughout Malaysia wear the same uniform. This policy has been implemented with the specific goal of promoting a sense of national unity and equity within an otherwise ethnically diverse country (Ministry of Education, 2013). In fact, the Malaysian education policy is structured based on this very goal to nurture a country that is ethnically integrate and a nation living in harmony and partnership (Ministry of Education, 2013). Unfortunately, until schooling age, Malaysian children often have limited exposure to members of different ethnic groups (e.g., Raman and Sua, 2010). Thus, the national school uniform is thought of as a key component of educational policies intended to establish a sense of similarity, community, and national identity among diverse ethnic groups (Spyrou, 2000). Some, however, have questioned the usefulness of uniforms in this regard (Jamison, 2006).

### Research Aim

Here, in three studies, we explored the experiences of former students, as well as teachers and school counselors in relation to Malaysia’s national school uniform policy. Respondents gave their impressions of current school uniform policy, its influence on student life, and whether it actually promotes school and national unity.

Study 1 investigated the perception and impression of national school uniforms amongst former Malaysian secondary school graduates using a self-report questionnaire. Study 2 and Study 3 used semi-structured interviews to collect the impressions of teachers and school counselors about the national school uniforms and their impact on students.

## Study 1

### Materials and Methods

#### Participants

One hundred and night two former Malaysian students (75 males, 117 females, *M*_age_ = 20.64 years, *SD* = 1.58) were recruited from college and university campuses. All participants were college or university students ranging from 18 to 23 years of age who had completed their primary and secondary education in a public or private secondary school in Malaysia. Participants came from four states: Johor, Selangor, Malacca, and Penang, as well as the Federal Territory of Kuala Lumpur. High school graduates were recruited rather than current high school students in order to avoid involving anyone under the age of 18 due to need for parental ethical consent. Permission was obtained from the participating colleges and university prior to data collection. Participation was completely anonymous. No identifying information was collected as part of the survey. Each participant was given RM5 as a token of appreciation for their involvement.

#### Design

The survey included both closed and open-ended questions. Means of closed-ended Likert-scale responses were used to gather overall ratings of students’ perceptions of school uniforms and its relation to school cohesion. Two open-ended questions were analyzed using inductive content analysis in a search for underlying themes. All questionnaires were given in English.

#### Materials

A survey which consisted of three parts were used for this study namely the demographical form, the School Uniform Perception questionnaire and Perceived Cohesion questionnaire.

##### School Uniform Perception Questionnaire (SUPQ)

The SUPQ is a self-developed questionnaire that asked participants to rate their school uniforms in terms of: Comfort, Attractiveness, Affordability, and Tidiness on a scale of 1–5 (1. *Strongly Disagree*–5. *Strongly Agree*. Sums of all ratings were obtained to suggest students’ overall perception of school uniform. Higher scores indicated more positive perception of the school uniform.

##### Perceived Cohesion Questionnaire (PCQ)

The PCQ is a self-developed questionnaire that included 13 closed-ended questions and 2 open-ended questions. Thirteen closed-ended Likert-type response questions (1. *Strongly Disagree*–5. *Strongly Agree*) asked students about perceived school cohesion (see Table 1). Sum of all ratings were obtained to suggest students’ overall perceived school cohesion. Higher scores indicated higher perceived social cohesion. Two open-ended questions gave participants the opportunity to express, in their own words, their levels of enjoyment and belonging in school generally and in relation to school uniforms.

**TABLE 1:** Items of the PCQ.

**Items**
1. Wearing school uniforms made all students equal 2. I felt a sense of belonging to my peers when I was wearing my school uniform compared to my casual wear 3. I liked being seen in my school uniform in public. 4. I preferred to engage with people who were from the same school and wore the same uniform as me. 5. I saw myself as part of the school whenever I was wearing my school uniform. 6. There was a strong sense of school spirit amongst the students in my school. 7. I saw myself as a committed member of the school. 8. My school uniform acted as a reminder for me that I was representing my school even when I was wearing them outside of the school. 9. I was excited about going to school. 10. I was happy to be studying in my school. 11. I felt proud to be representing my school in my school uniform 12. Overall wearing school uniforms gave me a sense of belonging toward my peers and school. Provide your reason(s) below 13. Overall, I enjoyed being in my school and I was happy to be in my school. Provide your reason(s) below

#### Ethics Statement

Approval was obtained from the Monash University Human Research Ethics Committee (Project #12135).

#### Procedure

192 participants were recruited through convenience sampling. Permission was obtained to conduct data collection from the participating college and university. Participants were approached in a classroom setting and asked if they were interested to participate. All participants read an Explanatory Statement before deciding whether or not to participate in the study. The statement explained that participation was completely anonymous and that they had the right to withdraw from the participation of the study at any stage. Participants were encouraged to respond openly and genuinely to all questions. Upon completion, participants were debriefed, dismissed and received RM5 upon submitting the questionnaire as a token of appreciation for their involvement.

#### Data Analysis

Data were screened for accuracy, missing values, and outliers. No missing values were found. All cases were examined for values that fell in the z-score limit of ±3.29 (Tabachnick and Fidell, 2007). A single outlier was identified through this process and deleted.

##### Reliability analysis

The internal consistency of the 4-items from the SUPQ and the 13-items PCQ were analyzed by examining the Cronbach’s alpha, respectively. The results of the reliability statistics showed good internal consistencies for both measures (see Tavakol and Dennick, 2011), with Cronbach’s alpha for SUPQ was α = 0.84 and PCQ was α = 0.89.

##### Factor analysis

A principal axis factor analysis was conducted on the 13 items in PCQ, with orthogonal rotation (varimax). The Kaiser-Meyer-Olkin (KMO) measure verified the sampling adequacy for the analysis, with KMO = 0.918, and all KMO values for individual items were greater than 0.77, which is well above the acceptable limit (Field, 2018). Additionally, Bartlett’s Test of Sphericity was significant supporting factorability of the matrix. An initial analysis was used to obtain eigenvalues for each factor in the data. Two factors had eigenvalues over Kaiser’s criterion of 1 and in combination explained 56.68% of the variance. The scree plot showed inflexions that justify retaining two factors. Both components revealed had high reliabilities, with Cronbach’s α = 0.89 for Component 1 (labeled as Sense of Belonging) and Cronbach’s α = 0.80 for Component 2 (labeled as Feelings of Morale).

##### Thematic analysis

Open-ended responses from the PCQ were analyzed using thematic analysis (Braun and Clarke, 2006). The analysis follows the six-phase guide as proposed by Braun and Clarke (2006): (1) Become familiar with the data, (2) Generate initial codes, (3) Search for themes, (4) Review themes, (5) Define themes and (6) Write-up. Transcripts were reviewed and final themes arrived at by consensus among all authors of this manuscript (Saldana, 2009; Hill(ed.), 2012).

### Results

The data was analyzed using the IBM SPSS Statistics 26 Software.

The level of agreement with each item is listed in Table 2. Students overall rated their uniforms as neutral to somewhat positive when it came to *tidiness, affordability*, and *comfort*. Students overall somewhat disagreed with the statement that their national school uniforms were *attractive*.

**TABLE 2:** Means and standard deviations of students’ agreeableness to each item.

	**Students’ perception of school uniform**	
	***M***	***SD***
Tidiness	3.79	0.84
Affordability	3.33	0.83
Comfort	3.06	1.02
Attractiveness	2.59	0.99

*Average scores on a scale of 1–5, where 5 is strongly agree (3 is neutral).*

A bivariate Pearson’s product-moment correlation coefficient (*r*) was calculated to assess the strength and direction of the relationship between students’ overall perception of school uniform and overall perceived cohesion. The correlation between the two variables was positive and statistically significant, *r*(190) = 0.74, *p* < 0.001. The results suggests more positive perception of the school uniform attributes to higher perceived cohesion amongst students.

#### Open-Ended Responses From Students

Content analysis was conducted to understand students’ experience with school uniforms and school cohesion based on their own words. Students’ perception on school cohesion will be explored based on their perceived sense of belonging and feelings of morale, the two factors identified to conceptualize perceived school cohesion.

##### Open-ended responses regarding school uniforms and sense of belonging

Three major themes were identified from the open-ended responses namely equality, representation of identity and school experiences, and the sense of community.

###### Equality

Some students (43/192) spoke of feelings of similarity and equality due to its standardization. The overall consensus of open-ended responses suggested by that wearing the same school uniform lessened the importance of differences in background factors such as socio-economic status thus making it easier to interact.

###### Representation of identity and school experiences

For many students (50/192), the school uniform simply highlighted the fact that they were students. Wearing school uniforms made them, and anyone observing them, keenly aware of their status as students. In this way, students felt differentiated from the rest of society. Notably, in the context of the government’s stated goal of establishing national unity, students cited the fact that badges differentiated specific schools from each other. Students felt automatically identified and grouped because of their school, and this was not necessarily a positive experience. Students from more reputable schools felt pride in their uniforms whereas others felt the opposite. Some students also cited feelings of restriction and limited autonomy because they were “forced” to dress in the same way as their peers. Wearing the school uniform for many was a matter of following regulations, not something which enhanced their sense of belonging.

###### Sense of community

Students (33/192) also expressed that the standardized appearance of the school uniform lessened the importance of differences, such as socioeconomic status, within the school. It also clearly showed the specific school that students attended. So, in this way it highlighted differences between schools.

##### Open-ended responses regarding feelings of morale toward school

Responses related to school morale, categorized here as positive and negative school experiences, generally did not relate to school uniforms.

###### Positive school experiences

A majority of students (121/192) who took the survey had positive feelings about their school and peers. Students expressed that they had good memories and experiences in school overall. The most common type of response involved positive experiences with friends and engaging with peers in school. Many students also expressed positive feelings about their teachers and the learning environment.

###### Negative school experiences

Students (71/192) who described negative school experiences cited an excess of rules and regulations, as well as sub-optimal teaching and peer-relations.

## Study 2

### Materials and Methods

#### Participants

Study 2 consisted of interviews with 10 secondary school teachers. Teachers were recruited in this study as they are the one of the key stakeholders of schools whose roles involve close engagement with students and understanding the development of student lives’ within the school environment (McCaleb, 2013). All participants were citizens of Malaysia who had worked as teachers for at least 3 years. Participants were recruited through existing contacts as well as referrals from other participants (snowballing).

#### Design

Semi-structured interviews were conducted with all participants. All interviews were carried out in English. Back translation was done with native Bahasa Malaysia speakers to assess the quality of translation done by the researcher (Tyupa, 2011). The interview consisted of 10 questions, which are listed in the Table 3.

**TABLE 3:** Interview questions for teachers and school counselors.

**Interview questions**
1. What are your views on national school uniforms? 2. What are the advantages and disadvantages of having a national school uniform? 3. Do you think students would share your views on the above? 4. Can you comment on some of the permitted variations of the national school uniform? 5. What are your views on the strengths and weaknesses of the permitted variations to the national school uniforms? • Any instances where students attempted to make variations to the uniform that was not permitted? 6. Do you think the following variations should be permitted? • A non-Muslim girl wants to wear *baju kurung*; a Muslim girl not wanting to wear the *hijab*, or wanting to wear the pinafore? • A non-Muslim boy wants to wear/play with a *songkok*; a Muslim boy not wanting to wear a *songkok*? 7. In your experience, do you think the national school uniforms help foster national identity? 8. Do you think the local community’s culture shapes policies on national school uniforms? 9. Have you noticed any changes in people’s perceptions of how to wear the school uniform? 10. Is there anything else you think is relevant to this discussion?

#### Ethics Statement

Approval was obtained from the Monash University Human Research Ethics Committee (Project #8391).

#### Procedure

Data collection started by contacting potential participants via telephone, email, or in person. All participants read an explanatory statement that described the project and outlined the guidelines for confidentiality and the participants’ role. The 10 participants who agreed to partake in the study signed a consent form, agreeing to be interviewed and have their responses audio recorded.

One-to-one interviews were conducted in a neutral location; all by the same researcher. All interviews were audio recorded, transcribed, and labeled with pseudonyms to preserve anonymity. No identifying information was stored with the data.

#### Data Analysis

All audio recorded interviews were transcribed for thematic content analysis. Thematic analysis was conducted using Braun and Clarke’s (2006) six-phase guide: (1) Become familiar with the data, (2) Generate initial codes, (3) Search for themes, (4) Review themes, (5) Define themes and (6) Write-up. Transcripts were reviewed and final themes arrived at by consensus among all authors of this manuscript (Saldana, 2009; Hill(ed.), 2012).

### Results

Responses were grouped into six general categories as described below.

1.**Advantages of National School Uniforms**

**Teachers’ views**: Overall, the teachers interviewed suggested that the benefits of national school uniforms fell into two categories: equality and student management.

**Equality**: All teachers agreed that uniforms were a positive in that they equalized students, and thereby limited instances of discrimination or bullying related to socioeconomic status.

Some teachers (4/10) suggested that school uniforms were important for safety and security. Specifically, students are easily distinguished from visitors on the school grounds. As one of the teachers mentioned: “…we just look at the student, we know that they are students…” Similarly, students who were in restricted places or involved in misconduct were easy to spot because of their uniforms.

While almost all teachers agreed that a benefit of uniforms was equalizing students, one teacher suggested that differences in socioeconomic backgrounds are still visible. She described “branded” uniform items (school bags, shoes) which are used to indicate economic privilege. Furthermore, she went on to question if equalizing students is really of benefit in the long run:

“…are we shielding our children from the harsh relative- realities of the world? When they come out to work, they’ll begin to realize that, hey, it is true you know, some people get to wear you know, Ferragamo shoes and some wear Bata. And we all work in the same office, you know and all of us, we earn different levels. Why is it that we all don’t wear the same clothes? Or why don’t we all drive the same cars?” (P04)

Teachers expressed concern about the types of clothing students would wear if there were no uniforms. They felt confident that students would dress “decently” in their uniforms as they provide clear guidelines and a standardized appearance. Teachers appreciated the role that uniforms played in helping keep students looking neat, tidy, and professional.

**Student Management**: All teachers suggested that the school uniforms help control and manage the students. The word “control” was used frequently by teachers. One teacher described uniforms as “weapons to educate” students to abide to the school rules, to be disciplined and well-mannered. Some also felt that students are more likely to identify with their school when wearing a uniform, and thus would make more effort to behave and act responsibly.

Teachers also suggested that the uniforms provided a sense of belonging in relation to the school; that students “feel like a big family” with uniforms being a “unifying thing, so, *rasa macam bersepadu lah*.” which loosely translates to “feeling integrated.” Again, this seems to relate to the idea of limiting comparisons and competition among students, thereby creating a more harmonious atmosphere.

**Perceived students’ views**: Almost all teachers agreed that students probably did not like school uniforms. One teacher mentioned the idea of not having to find or think about different clothing to wear every day. But she also suggested, based on her own experience, that students really only appreciate this after they leave school. Others mentioned that school uniforms could help parents to save money and time because they don’t feel pressure to buy new or expensive outfits for their children.

2. **Disadvantages of National School Uniforms**

**Teachers’ views**: While most teachers felt that national school uniforms are beneficial overall, they did bring up some issues. Again, the idea that students often find them “annoying,” came up. Uniforms, being standard, often may not work as well with different body types. Also, the white uniforms can easily become stained by sweat and tend to retain body odor and, in heavy rain, white shirts may become partially transparent. Thus the uniforms themselves can, at times, become sources of self-consciousness and social discomfort.

Teachers mentioned sometimes needing to enforce uniform-related rules, which can take time and effort away from other school issues. Others, however, made the opposite point; that having uniforms makes the issue of clothing less salient, thus removing potential distractions.

**Perceived students’ views**: Most teachers agreed that students do not like the uniform and that it reduces student autonomy and creativity. As one teacher suggested, “we are teaching students to follow and conform.” Even permitted variations are defined by rules. Teachers suggested overall that, given a choice, students would prefer to choose their own clothes. One suggested that the “bold” or “fashionable” students, in particular, tend to dislike uniforms, but others do not mind them so much. Others echoed this sentiment; “some might like to be fashionable…I think those who are poor, they prefer to have the uniform, save a lot of money…”

Again, some more specific criticisms of the uniforms came up in relation to teachers’ understanding of students’ experience with uniforms. One issue was weather-appropriateness. According to teachers, students complain that the uniforms have too many layers and are stifling in hot weather. Others suggested that the short skirt makes some girls self-conscious, especially when sitting cross-legged. While students can opt for a *baju kurung* (traditional longer dress) instead, it tends to be constricting. Again, the transparency of the white shirts was also mentioned.

3. **Cultural/Religious Variations**

Most teachers discussed differences between non-Muslim and Muslim female students in regard to uniform variations. Generally, Muslim students do not see the shorter skirt as an option. One teacher also suggested that more religious teachers often encourage non-Muslims students to choose the more conservative option as well:

“Yeah, it’s highly recommended for a non-Muslim to wear *baju kurung*, in fact, when you wear, you get brownie points, alright, meaning, you know, it will be, you know, you will be always glorified by the Malay teacher, Muslim teachers, *Ustazah, Ustaz*, they will glorify you…you know, they love it.” (P07)

One participant noted, however that non-Muslim female students were specifically not allowed to wear the *baju kurung* in their school. This particular school has a majority of Chinese students and the religious teachers use the *baju kurung* to identify Muslim female students.

Teachers reported that it is strongly encouraged for female Muslim students to wear the *baju kurung* in order “cover up.” Muslim students who do not want to wear the *baju kurung* are essentially “encouraged” until they change their mind. Similarly, female Muslim students who do not normally wear the hijab (headscarf), are sometimes made to do so in school. This is an issue that non-Muslim, or non-religious teachers essentially leave to religious teachers as it is a matter of cultural and religious sensitivity:

“…. this one always handled by the…the teacher that teach *Agama (religious studies), Ustaz*, yeah. So, we seldom…because it’s sensitive, right….” (P05)

All of the teachers appeared to accept the cultural and religious reasons underlying uniform variations. One participant discussed the importance of cultural sensitivity:

“I feel that in school we have to be decent. You’re with…you’re in a co-ed school, you need to be decent because there are people watching you, and… know…of course, …we’re an Islamic country, so, you know, we need to *tutup aurat* (cover up). Okay, of course, we don’t need to *tutup aurat*, but we need to conform to that, you know, which is fine with our culture anyway.” (P03)

Teachers agreed that religious variations ought to be accepted without resistance and reported that students seemed to understand them as well.

4. **Community/Environment**

Although the national school uniform is a manner of policy, different regions, and schools apply the policy differently. Teachers all suggested that school management was the main source of variation between schools. Regarding whether female Muslim students should be required to wear the hijab (head scarf), one participant said:

“For me, it’s like, there is no black and white and I think it’s difficult to have black and white on this issue because for example if you said, okay, all Muslim students starting from by age of 13, they have to start wearing hijab in school. I can imagine those… we call it the ultramodern Malays. I can imagine… because they are already many of them out there; even pointing on things which I feel no need to be addressed because it’s already stated clearly in Al Quran but this one will be a big issue. I think that’s why probably, *Kementerian* (Ministry of Education) feels that it’s up to the PPD, *Pejabat Pendidikan Daerah*; it’s up to the school management.” (P02)

In other words, it is each school’s principal that has the final say on which variations are acceptable and the degree of freedom that students are allowed.

Teachers also mentioned that, although the school principal has the final say, the local community, and related factors such as parental education and income status influence school policies and uniform variations. In some areas, it might be more acceptable for a Muslim student not to wear a hijab, depending on her “family culture.” Usually if the family does not see it as a problem, the school will not get involved. However, several teachers did point out cases where students do not wear the hijab at home, but were “encouraged” to wear it in the school by certain teachers. Some teachers expressed dismay at such practices, but again cited religious sensitivities as reasons not to involve themselves in such decisions. Overall, teachers reported that schools in urban areas tended to be more open regarding uniform variations compared to those in more rural areas.

5. **National Social Cohesion**

All teacher participants agreed that the national school uniform policy has *no* relation to national identity or feelings of national unity. They also agreed that uniforms were essentially a positive thing overall.

Teachers felt that uniforms related more closely to local, within-school relationships. For example, one teacher reported:

“Actually, unity is from the school, then only come to country… if the school cannot unite the students, among the races, how can the country? The most important is from the school. It begins from the school. So, the teacher is very important. From school, you teach morals, you teach about the sense of unity… So, with the right input to the students, you will build up the correct thinking.” (P02)

Another teacher gave this example:

“We have been wearing that uniform for so many years, and yet there are, racial issues from time to time, and you know, fights among boys, over, what can be seen racial based, problems… they may start off with calling names, and making inappropriate remarks, more to that, not… I mean, they are wearing their school uniform and yet they are able to… instigate fights, you know, this kind of thing, and so, I think uniform has nothing to do with national cohesion.” (P01)

Again, although teachers did not feel that national school uniforms contributed to national social cohesion, they did report that the uniforms are an accepted norm throughout the country, and that they have other benefits.

6. **Changes to the Uniform**

Some teachers felt that, for many girls, a pants option would be better in terms of comfort, decency, and safety. Some also suggested alternative color options. Most teachers, however, felt that overall the uniforms did not need to be changed.

## Study 3

### Materials and Methods

#### Participants

Study 3 consisted of semi-structured interviews with six secondary school counselors who had at least 3 years’ experience at working at government schools. School counselors were recruited in this study as they are known to be one of the key stakeholders of schools whose roles involve working cooperatively for the success of students and understanding the development of student lives’ within the school environment (Hernández and Seem, 2004). There were no other exclusion criteria. Counselors were recruited through personal contacts and snowball sampling.

#### Ethics Statement

Approval was obtained from the Monash University Human Research Ethics Committee (Project #8391).

#### Design

Semi-structured interviews were conducted with all participants. All interviews were carried out in English. Back translation was done with native Bahasa Malaysia speakers to assess the quality of translation done by the researcher (Tyupa, 2011). The interview consisted of 10 questions, which are listed in Table 3.

#### Procedure

Data collection started by contacting potential participants via telephone, email, or in person. All participants read an explanatory statement that described the project and outlined the guidelines for confidentiality and the participants’ role. The six participants who agreed to partake in the study signed a consent form, agreeing to be interviewed and have their responses audio recorded.

One-to-one interviews were conducted in a neutral location; all by the same researcher. All interviews were audio recorded, transcribed, and labeled with pseudonyms to preserve anonymity. No identifying information was stored with the data.

#### Data Analysis

All audio recorded interviews were transcribed for thematic content analysis. Thematic analysis was conducted using Braun and Clarke’s (2006) six-phase guide: (1) Become familiar with the data, (2) Generate initial codes, (3) Search for themes, (4) Review themes, (5) Define themes and (6) Write-up. Transcripts were reviewed and final themes arrived at by consensus among all authors of this manuscript (Saldana, 2009; Hill(ed.), 2012).

### Results

Four main themes were obtained from the interviews. These are described with examples below. Because English was not the first language of many participants, quotes may contain grammatical and vocabulary errors. Errors were left largely intact so as not to bias reporting and allow the reader to gain a better sense of the interview content.

1. **School Uniforms and National Social Cohesion**

While three out of six counselors agreed that it was possible that school uniforms could promote social cohesion, there was not a consensus as to how this might work. Two participants related uniforms to the establishment of a national language for education (Bahasa Melayu – the Malay language, as opposed to English and Chinese which are also widely spoken, is the official language of public education in Malaysia). They suggested that standardizing uniforms might create a sense of unity, similar to how a national language is thought to bring about a greater sense of commonality.

Some counselors (3/6) discussed that differing rules and expectations for non-Muslim and Muslim female students detract from the creation of unity. Generally, although non-Muslim female students may choose between wearing a pinafore (skirt-style dress) and the *baju kurung* (a long tunic that is traditional clothing for Malay women), Muslim girls are expected to wear the *baju kurung* (There is only one uniform option for secondary school boys). Counselors felt overall that it would be better to have single female uniform, but this is problematic because in most regions the *baju karung* is associated with Malay (i.e., Muslim) culture and thus forcing non-Muslims to wear it could be seen as a kind of religious imposition. Changing to some other style of uniform was also seen as problematic because it would be difficult for stakeholders to agree on a single option that was acceptable to all.

2. **Religious/Racial/Ethnic Considerations**

Counselors discussed the complications related to religious diversity extensively in relation to school uniforms. Essentially, religious guidelines for female dress limit the options for most Malay-Muslims and, although other types of religious symbols are often allowed as uniform alterations, there was a great deal of inconsistency in allowances and restrictions between schools.

3. **Student Views**

Counselors suggested that overall Malaysian students dislike their current uniforms. Specifically, they mentioned a lack of choices for Muslim females, as well as a general lack of comfortability and attractiveness. Counselors explained students’ tendency to both overtly and subtly modify their uniforms as a reflection of this.

4. **Counselors’ View of Current Implementation of School Uniforms**

Overall the counselors felt that school uniforms were good for students, citing their practicality and convenience. They generally felt that the current uniform design required few or no changes, but they were not opposed to allowing some variations, such as in color rather than design, in different circumstances.

## General Discussion

This research set out to understand the attitudes of Malaysian high school students, teachers, and school counselors toward the national school uniform. As a corollary we were interested in whether school uniforms contribute to the stated goal of promoting cohesion or group identity on the national or school level.

Overall, students were neutral to somewhat positive about the national school uniforms in terms of tidiness, affordability and comfort, however, they did not find them to be attractive. Those who had positive perceptions of their uniforms and also tended to have higher levels of school loyalty. Also, a majority of students felt that uniforms led to greater equality by lessening the salience of socioeconomic differences. These findings showed consistency to existing research on school uniform as a mechanism for establishing deindividuation, respect and loyalty toward their school and its stakeholders (Craik, 2003). Student did not feel, however, that uniforms contributed to positive feelings toward school in general, reporting that other factors such as friendship quality, teacher-student interactions, and school environment were more important.

Generally, the views of teachers and counselors did not contradict those of students. They did not see uniforms as of much use in promoting national identity, but they did see uniforms as a means of minimizing socioeconomic differences as well as reducing the level of distractions overall. Teachers and counselors both saw uniforms as a tool for promoting discipline, safety, and conformity within the school environment. These findings showed consistency with Baumann and Hana’s (2016) study which found that the implementation of school uniforms in schools limits any form of competition over appearance among students which could result to distractions in class, and disciplinary issues like stealing and bullying.

Overall, students, teachers and school counselors were accepting and moderately positive toward the national school uniform policy, although, contrary to the intended implication, their sentiments were not related to the intrinsic feelings of greater school and national level cohesion. Rather, the national school uniform implicate the purpose of maintaining control and discipline amongst student as well as a mean to narrow the discrepancy in appearance and socioeconomic differences amongst students and family backgrounds. There were several concerns about the practicalities of the school uniform designs, especially for female students (white color blouse and *baju kurung*), that may contribute to the dislikes and discomforts of wearing them on a daily basis in Malaysia’s humid climate. Other related factors such as the varying regulation of school managements as well as cultural and religious requirements also come into play in determining the way school uniforms are worn in schools.

### Recommendations for Future Uniform Policy

Despite an overall acceptance of uniforms in general, something of a consensus arose among student, teacher, and counselor responses in regard to changes that might improve upon the policy. More options for females, particularly Muslims were suggested; perhaps different color blouses or a pants option, were suggested as ways of increasing cultural inclusivity in the school uniforms as well as allowing greater flexibility. Occasional days where students are allowed to choose what they would like to wear while abiding by a certain dress code (i.e., formal wear, traditional wear) was also suggested.

### Limitations and Recommendation for Future Study

A limitation that is worth highlighting in this study was the use of non-representative sampling. The current study is reliant on the retrospective recollection of public school graduates as well as insights of teachers and school counselors’ observation and insights of students’ perception. Although this method provided essential understanding from key stakeholders about the nature of the cohesion within the school setting, it still lacks actual, more objective perspectives of the topic which can be obtained through recruiting a more representative sample group, which is current high school students themselves. Also, the number of teacher and school counselor participant was relatively small and they were only drawn from the more urban areas of Selangor and Kuala Lumpur. A more representative sample would include participants from a broader cross-section of regions, particularly more rural areas. Nevertheless, this study offers a unique perspective and insight into important characteristics that should be implemented within the national education policies in regards to rules about school uniforms.

## Conclusion

In conclusion, Malaysian students, teachers and school counselors in the current study had generally positive attitudes toward the national school uniform. Generally, teachers and counselors felt that the similarity in appearance helped to lessen the relevance of socioeconomic differences and promote the ease to govern students, ensuring discipline and safety. There were mixed responses in regard to the types of variations, or exceptions that should be allowed. Most teachers felt that uniforms, if anything, should be more standardized so as to limit student differences. However, teachers and counselors both discussed the challenges related to offering appropriate and acceptable options for both Muslim and non-Muslim females as well as how to deal with varying degrees of restriction within the Muslim community. Finally, in regard to school unity and identity, students, teachers and counselors agreed that, although school uniforms might make membership in a specific school body more salient, it is the school culture, and the quality of relationships among peers as well as between students and teachers that plays a much greater role. Similarly, overall, students, teachers, and counselors agreed that uniforms essentially played no role in promoting national identity. In fact, students pointed out that the various school badges, if anything, made them feel more different, or separate, from students of other schools rather than of the same group. Similarly, differences in uniform requirements for Muslims and non-Muslims may serve to highlight, rather than diminish the salience of religious differences. Thus, promoting national identity or cohesion in a multicultural society is an obviously complex task that school uniform policies may or may not be able to play a meaningful role in. Nevertheless, the overall attitudes of students, teachers and school counselors toward school uniforms are positive and suggest multiple benefits to the policy. For this reason, although some minor changes could be suggested, the national school uniform policy is relatively uncontroversial as it stands.

## Data Availability Statement

The datasets generated for this study are available on request to the corresponding author.

## Ethics Statement

Approval was obtained from the Monash University Human Research Ethics Committee (MUHREC – Projects #12135 and #8391).

## Author Contributions

All authors contributed to the design and presentation of this study.

## Conflict of Interest

The authors declare that the research was conducted in the absence of any commercial or financial relationships that could be construed as a potential conflict of interest.
